# Does Calorie Restriction Modulate Inflammaging via FoxO Transcription Factors?

**DOI:** 10.3390/nu12071959

**Published:** 2020-06-30

**Authors:** Sang-Eun Kim, Ryoichi Mori, Isao Shimokawa

**Affiliations:** Department of Pathology, School of Medicine and Graduate School of Biomedical Sciences, Nagasaki University, 1-12-4 Sakamoto, Nagasaki 852-8523, Japan; kim-se@nagasaki-u.ac.jp (S.-E.K.); ryoichi@nagasaki-u.ac.jp (R.M.)

**Keywords:** calorie restriction, FoxO, inflammaging, NLRP3 inflammasome, cellular senescence

## Abstract

Calorie restriction (CR) has been shown to extend lifespan and retard aging-related functional decline in animals. Previously, we found that the anti-neoplastic and lifespan-extending effects of CR in mice are regulated by forkhead box O transcription factors (FoxO1 and FoxO3), located downstream of growth hormone (GH)–insulin-like growth factor (IGF)-1 signaling, in an isoform-specific manner. Inflammaging is a term coined to represent that persistent low-level of inflammation underlies the progression of aging and related diseases. Attenuation of inflammaging in the body may underlie the effects of CR. Recent studies have also identified cellular senescence and activation of the nucleotide-binding domain, leucine-rich-containing family, pyrin-domain-containing-3 (NLRP3) inflammasome as causative factors of inflammaging. In this paper, we reviewed the current knowledge of the molecular mechanisms linking the effects of CR with the formation of inflammasomes, particularly focusing on possible relations with FoxO3. Inflammation in the brain that affects adult neurogenesis and lifespan was also reviewed as evidence of inflammaging. A recent progress of microRNA research was described as regulatory circuits of initiation and propagation of inflammaging. Finally, we briefly introduced our preliminary results obtained from the mouse models, in which *Foxo1* and *Foxo3* genes were conditionally knocked out in the myeloid cell lineage.

## 1. Introduction

Aging, an inherent risk factor for noncommunicable diseases such as cardiovascular and neurodegenerative diseases, has been a major burden not only for individuals but also at the societal level, owing to the rapidly increasing costs of medical and nursing care associated with the aging of society [1]. Understanding fundamental mechanisms of the biology of aging and applying such knowledge to retard aging have become increasingly important in recent decades.

In a range of organisms including nonhuman primates, calorie restriction (CR) regimens, which limit dietary intake to 60–80% of that in control organisms fed ad libitum (AL), can extend the average and/or maximum lifespan, when compared with that of AL controls [2,3]. CR regimens have also been shown to prevent aging-related diseases. Other studies have demonstrated that insulin-like growth factor 1 (IGF-1) signaling is a common factor regulating aging and the lifespan [4,5,6,7]. Because CR reduces IGF-1 signaling, it has been considered to be one of the key signals by which CR retards aging. Recent studies have identified activation of forkhead homeobox type O (FoxOs) transcription factors and inhibition of mechanistic target of rapamycin (mTOR) as promoters of longevity downstream of the IGF-1 signal, besides the effects of CR [6,8].

Inflammaging is a term coined to represent chronic low-level inflammation underlying the progression of aging [9]. Since Franceschi et al. proposed the concept in 2000 [9], this term has been frequently used in the research field of biomedical gerontology [10,11,12,13]. During the aging process, innate immunity is activated, in contrast to adaptive immunity, which is suppressed. This relative activation of innate immunity results in the propagation of inflammatory responses, leading to chronic low-level inflammatory phenotypes in aging tissue [9,14]. This is also referred to as senoinflammation [15].

In this paper, we review growing evidence indicating the mechanisms underlying the initiation and propagation of inflammaging, with special reference to senescence-associated secretory phenotype (SASP) [16] and inflammasome activation. The involvement of FoxO3 and microRNA (miRNA) in inflammaging is also discussed with regard to the salutary effects of CR. Finally, we present our preliminary data supporting the hypothesis that CR exerts its effects via the inhibition of inflammaging.

## 2. Chronic Low-Level Inflammation and Aging

Chronic, sterile, low-level inflammation has been considered as a factor that accelerates the aging process, which is referred to as “inflammaging” [9,17] or “senoinflammation” [15]. This persistent low-level inflammation conferring toxic insults on tissues not only accelerates aging but also causes aging-related diseases.

Cellular senescence, primarily reported as a phenomenon in vitro [18], has been shown to occur in various tissues of animals [16]. Recent studies have demonstrated that senescent cells display a phenotype called SASP, which is characterized by the release of inflammatory cytokines, chemokines, matrix metalloproteases (MMPs), and growth factors [19]. In other words, senescent cells are thought to be not merely non-proliferating quiescent cells but instead to cause or accelerate aging and related disorders by eliciting inflammation in aging tissues. By contrast, experimental elimination of senescent cells has been reported to effectively relieve the symptoms or delay the progression of several disease models [20,21,22]. Senescent cells are currently compelling targets for preventing and treating aging and related disorders.

Macrophages have critical roles in not only inflammatory responses but also in maintaining metabolic homeostasis and regenerative responses [23]. In aged mice, selective removal of macrophages is reported to mitigate peripheral neurodegeneration and muscle weakness [24]. Depletion of macrophages in aged mice has also been shown to improve survival against a systemic inflammatory challenge, in contrast to the case in young mice [25]. Hall et al. reported that macrophages highly expressing cellular senescence markers (p16^INK4a^ and β-galactosidase) accumulate around senescent cells implanted in tissues even in young mice [26]. Taking these findings together, macrophages, activated by senescent cells or even by some other mechanisms, could play major roles in persistent low-level inflammation in the aging process, namely, inflammaging [27].

## 3. Activation of Inflammasome in the Aging Process

Although chronic low-level inflammation was recognized as being linked to aging and related diseases, the molecular mechanisms that elicit inflammation in aging tissues were largely unknown. A couple of decades ago, inflammasomes were identified as cytosolic multiprotein complexes, which are critical mediators of inflammatory processes [28]. Inflammasomes recognize microbial components and injured-cell-derived molecules such as adenosine triphosphate (ATP), uric acid, oxidatively modified DNA, and aggregated proteins. These are called pathogen-associated molecular pattern molecules (PAMPs) and danger-associated molecular pattern molecules (DAMPs), respectively [29]. 

Nucleotide-binding domain, leucine-rich-containing family, pyrin-domain-containing-3 (NLRP3) inflammasome is well characterized as a distinctive innate immunosensor that can be activated by DAMPs [29,30,31]. When cells are exposed to harmful stimuli or stressful conditions, such as toxic chemicals or hypoxia, they suffer damage and secrete DAMPs [32]. The process of NLRP3 inflammasome activation comprises priming and activating steps. During the priming step, DAMPs are recognized by toll-like receptors (TLRs), which leads to NF-ĸB activation and induction of the expression of target genes including *Interleukin 1b (Il-1b)*, *Il-18*, and *NLR family, pyrin domain containing 3 (Nlrp3).* During the activation step, NLRP3 binds to apoptosis-associated speck-like protein (ASC), which recruits pro-caspase-1 to form the NLRP3 inflammasome complex. Inflammasome assembly leads to the activation of pro-caspase-1 by self-cleavage, and then this activated caspase-1 converts pro-IL-1β and pro-IL-18 to their respective active forms. Biologically active IL-1β and IL-18 elicit sequential inflammatory responses, leading to pyroptotic cell death (pyroptosis).

A study using a *Nlrp3* gene-deficient mouse model showed that NLRP3 activation is associated with age-related inflammation and dysfunction in adipose tissue, brain, and thymus [32], supporting the connection between inflammaging and NLRP3 inflammasome activation.

## 4. Inflammaging in the Brain

### 4.1. Inflammaging in Neural Stem/Progenitor Cells

The aging brain displays reduced neurogenesis, increased synaptic aberrations, higher metabolic stress, and augmented inflammation [33]. These alterations cause cognitive decline and neurobehavioral deficits in humans.

In rodents, adult neurogenesis persists in the subgranular zone (SGZ) of the hippocampus, the ventricular and subventricular zone (SVZ) of the lateral ventricle, and along the third ventricle and the mediobasal hypothalamus (MBH) [34,35]. Although the production of new neurons diminishes dramatically with age, newly generated neurons are involved in maintaining brain functions. For example, newly formed neurons in the hippocampus play roles in learning, memory, and pattern separation [36], and thus the dysregulation of adult neurogenesis could accelerate brain aging and may promote neurodegenerative disorders. Experimentally, it has also been reported that neural stem or progenitor cells (NSCs or NPCs) in the MBH control metabolic and neurobehavioral deficits and lifespan in mice [37,38,39].

Growing evidence has indicated that excess energy intake promotes inflammation and impairs neurogenesis in the hypothalamus. High-fat-diet (HFD) feeding of mice in the neonatal and adolescent periods promotes neurogenesis in the median eminence of the hypothalamus [40]. HFD feeding also decreases the number of neural stem cells in the mediobasal hypothalamus (MBH) and impairs the neuronal differentiation ability of NSCs by activating the nuclear factor kB (NF-ĸB) pathway [37].

Dongsheng Cai’s group reported that the NF-ĸB pathway is activated in the hypothalamus, and that inhibition of this activation by delivering a gene encoding dominant-negative (DN) inhibitor of kappa B (IĸB)-α in the MBH in middle-aged to elderly mice extended the lifespan [38]. By contrast, delivery of a gene encoding constitutively active IĸB kinase beta (IKK-β), which activates the NF-ĸB pathway in the BMH, shortened the lifespan. In these mouse models, aging-related deteriorations such as reduced size of muscle fibers, reduced dermal thickness, reduced bone mass, and increased tendon stiffness were improved. They also exhibited aging-related activation of microglia, which release Tumor necrosis factor-alpha (TNF-α) during the early phase of aging, as a causative factor of aging.

Hypothalamic-microglia-specific knockout of the *IKK*-*β* gene ameliorated the aging-related physiological changes. Furthermore, neuron-specific inhibition of IKK-β extended the lifespan. A subsequent mouse study, in which hypothalamic NSCs were genetically ablated, showed that the mice displayed a shortened lifespan with impairments of functions such as muscle endurance, coordination, and treadmill performance [39]. By contrast, hypothalamic implantation of DN-IĸB-α-expressing-NSCs extended the lifespan in mice with improvements of the aging-related declines in neurobehavioral activities. This series of experiments by Dongsheng Cai’s group clearly indicated the substantial role of hypothalamic NSCs in regulating aging and lifespan and that aging is accelerated by inflammation initiated by microglia [37,38,39].

The progression of neurodegenerative diseases such as Parkinson’s disease (PD) is attributable to impairments of adult neurogenesis in the SVZ [41]. An *α*-*synuclein* (*Snca*) mutant transgenic mouse model of PD showed decreases in the numbers of NSCs and/or NPCs in the SVZ at 5 to 6 months of age [42]. In that study, mutant Snca promoted activation of the NLRP3 inflammasome in NSCs; by contrast, knockout of the *caspase-1* gene ameliorated the inhibition of adult neurogenesis by mutant *Snca.* These findings indicate that inflammasome activation suppresses the proliferation and differentiation of NSCs/NPCs in the SVZ. 

Either inhibition of the priming step, namely, activation of NF-ĸB, or disruption of the activation step of NLRP3 inflammasome by the deletion of *caspase-1* improves the impairment of adult neurogenesis. It remains to be elucidated whether CR inhibits inflammation via FoxO3, and then maintains the pool of NSCs in old age.

### 4.2. Effects of CR on Adult Neurogenesis: An Implication of FoxO3

CR is reported to improve age-related declines in brain functions and inhibit many models of neurodegenerative diseases [43,44]. Therefore, we can surmise that CR maintains adult neurogenesis in an appropriate state later in life. Indeed, accumulating evidence supports this hypothesis [34]. In particular, aging-related atrophy of the gray matter in subcortical regions of the brain in rhesus monkeys subjected to 30% CR was reported to be slowed compared with that of AL controls [45].

The effect of CR on adult neurogenesis has been tested in rats and mice subjected to different CR regimens. Published data indicate beneficial effects of CR on adult neurogenesis, such as enhanced survival of neuroblasts or newly formed neurons, although the details of the findings differ somewhat among those studies, probably due to the variety of experimental settings, such as the age, strain, or sex of the mice subjected to the pulse–chase approach [46,47,48,49,50]. To our knowledge, there are limited data directly indicating that adult neurogenesis persists or that the NSC pool is maintained in old age in rodent models by CR, probably due to technical limitations of the pulse-labeling and chase approach with thymidine analogs to detect very low levels of proliferation of NSCs or NPCs. However, notably, Apple et al. [50] reported that CR protects against aging-related loss of immunostained areas with double cortin (DCX), a neuroblast marker, in the SVZ. They related this finding to the protective effect of CR on aging-related acceleration of inflammatory microenvironments in the SVZ. The number and area of microglia immunostained by the Iba-1 antibody were shown to be significantly greater in aged AL mice than in age-matched CR mice [50].

Even haploinsufficiency of the *Foxo3* gene diminishes the life-extending effect of CR in male C57BL6/J mice [51], indicating a substantial role for FoxO3 in the effect of CR. CR may affect adult neurogenesis via FoxO3 and then improve or protect against the age-related decline of brain functions. Renault et al. [52] indicated that the NSC population is decreased in *Foxo3*^−/−^ mice compared with that in wild-type mice at young and middle ages but not at the neonatal stage. The NSC population was estimated based on the number of BrdU-labeled cells among SGZ cells using the pulse–chase approach and the neurosphere-forming ability of NSCs isolated from forebrain tissues. The neurosphere assay also indicated that the ability of NSCs to generate neural lineages is defective in *Foxo3*^−/−^ mice. The neurosphere experiments suggest that FoxO3 inhibits the G_1_/S transition of NSCs in the cell cycle, and thus suppresses the excess proliferation of NSCs and NPCs. The loss of FoxO3 could result in loss of the ability of NSCs to re-enter the quiescent state after they divide, leading to the amplification of progenitors and exhaustion of the pool of NSCs in vivo. As a result, the brain becomes heavier in *Foxo3^−/−^* than in wild-type (WT) mice [52]. Paik et al. [53] also reported that *Foxo1*-, *3*-, and *4*-deficient mice show an initial increase of proliferation of NSCs/progenitor cells during early postnatal life, but this is followed by a precocious decline in the NSC pool and accompanying neurogenesis in the adult brain. They used triple *Foxo (1, 3, and 4*)-null mice because germline or conditional knockout of each gene or combinations of two genes caused minor changes in the NSC pool or neurogenesis until middle age in mice. Nonetheless, they also stated that FoxO3 has the greatest phenotypic impact, particularly regarding the increased brain size. In contrast, overexpression of constitutively active FoxO3 in the forebrain reduces the brain size due to the apoptotic loss of NPCs during the embryonic stage and young adulthood in mice [54]. A negative regulator of class Ia phosphatidylinositol-3-kinase (*Pik3ip1*), which is a FoxO3-target gene, has been highlighted as a causative factor for the enhanced apoptotic activity in transgenic mice [54]. Collectively, these findings suggest that the germline loss of the *Foxo3* gene results in excess growth of the brain via promoting the proliferation and neuronal differentiation of NSCs/NPCs, as well as attenuating the removal of neuronal cells by apoptosis.

The above-mentioned studies of FoxO3 in adult neurogenesis were conducted in mice kept under standard AL conditions. However, our lifespan study indicated a substantial role for FoxO3 under CR conditions [51]. In our lifespan study, the brain weight in *Foxo3*^+/−^ CR mice was significantly greater than that in wild-type (WT) CR mice in middle age (Supplemental Figure S1); the brain weight in *Foxo3*^+/−^ CR mice did not differ from that in WT-AL mice. Therefore, we surmise that the reduced FoxO3 expression promotes brain growth even under CR conditions, supporting the involvement of FoxO3 in the effect of CR on neurogenesis.

Limited data are available indicating the involvement of FoxO3 in the effect of CR on brain aging, particularly the suppression of neuroinflammation. It remains to be elucidated whether CR inhibits inflammation via FoxO3, and then maintains the pool of NSCs in old age.

## 5. Modulation of Inflammaging by Calorie Restriction (CR) via NLRP3 Inflammasome: A Possible Connection with FoxOs

CR is reported to effectively reduce circulating inflammatory signatures that are induced by aging or obesity [55,56,57]. The molecular mechanisms by which CR affects aging and related diseases are well reviewed elsewhere under the concept of “senoinflammation” [15]. Several studies have already indicated the inhibitory effect of CR on the aging-related increase of inflammation. 

A recent study reported that the anti-inflammatory effects of CR or ketogenic diets, potentially CR-mimetic diets, may be related to the NLRP3 inflammasome [58]. Fasting was shown to reduce NLRP3 inflammasome activation in macrophages in a human fasting/refeeding study [59]. Furthermore, fasting for 48 h has been reported to suppress the NLRP3 inflammasome in a sirtuin 3 (Sirt3)-dependent manner [60]. By contrast, Ming et al. reported that the NLRP3 inflammasome was particularly activated in macrophages by six-month-long feeding of a high-fat diet in young mice and two-year-long feeding of a standard diet in mice [61]. Thus, dietary energy intakes could affect activation of the NLRP3 inflammasome.

IGF-1 signaling is now recognized as an evolutionarily conserved pathway that regulates growth and aging/lifespan in a pleiotropic manner [5]. The growth hormone (GH)–IGF-1 axis is well known to promote development and growth in vertebrates; by contrast, reduced GH–IGF-1 signaling results in dwarfism but extends the lifespan and retards the aging process. The lifespan extension in nematodes by the reduction of insulin-like signaling requires the activity of the *daf*-*16* gene [62], which corresponds to the *Foxo* genes in mammals [63]. Subsequent studies using invertebrate or vertebrate models confirmed that the reduction of IGF-1 signal consistently increases the lifespan compared with that in wild-type control animals [64,65], and that the family of FoxOs is involved in extension of the lifespan in a range of organisms [65,66].

It was predicted that CR exerts its effects by reducing the IGF-1 signal, and thus FoxOs may be required for the effects of CR. Although it depends on the culture conditions, the necessity of Daf-16 for the life-extending effect of CR has been proven using *daf-16* mutants [67]. Our lifespan studies in mice also suggested that FoxOs play pivotal roles in the effects of CR [51,68]. Briefly, haploinsufficiency of FoxO1 (*Foxo1*^+/−^) abated the anti-neoplastic effect of CR, whereas the decline of FoxO1 did not alter the CR-induced lifespan extension [68]. Moreover, using *Foxo3*^+/−^ and *Foxo3*^−/−^ mice, we reported that FoxO3 contributed most to the life-extending effect of CR [51]; in other words, the life-extending effect of CR was diminished in *Foxo3*^+/−^ and *Foxo3*^−/−^ mice. However, FoxO3 might not affect the anti-neoplastic effect of CR, unlike FoxO1 [51].

FoxO transcription factors, which are highly conserved in different species, are involved in the regulation of multifaceted cellular and physiological functions including cell growth, differentiation, apoptosis, stress response, DNA repair, and mitochondrial bioenergetics [69]. Recent studies have indicated that FoxO3 is involved in the inhibition of SASP in senescent cells.

**Figure 1:**
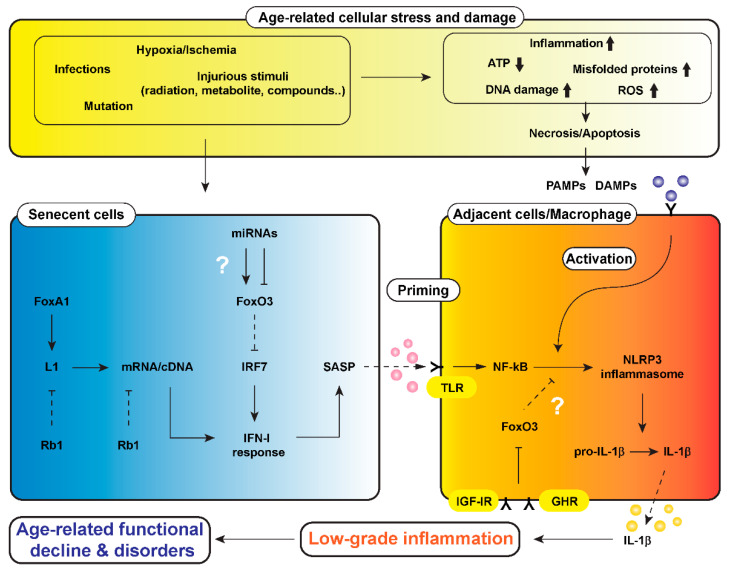
A hypothetical scheme of a mechanism underlying low-grade inflammation that causes age-related functional decline and disorders in the body, i.e., “inflammaging”. Cells are always exposed to stresses such as hypoxia/ischemia, injurious stimuli, mutations, and infections. These cellular stresses cause a reduction of adenosine triphosphate (ATP) generated in mitochondria or increments of reactive oxygen species (ROS), misfolded proteins, DNA damage, and inflammatory cell infiltration, leading to necrosis and/or apoptosis of cells. Injured-cell-derived molecules and microbial components, called damage-associated molecular patterns (DAMPs) and pathogen-associated molecular pattern (PAMPs), are involved in activation of the nucleotide-binding domain, leucine-rich-containing family, pyrin-domain-containing-3 (NLRP3) inflammasome in macrophages and parenchymal cells. Cellular stresses also increase senescent cells, in which the long-interspersed element-1 (L1) is activated, following by induction of type 1 interferon (IFN-I) response and senescence-associated secretory phenotype (SASP). The SASP primes through toll-like receptor (TLR) and activates NLRP3 inflammasome in cells adjacent to senescent cells. FoxO3 could be a target for calorie restriction (CR) and growth hormone (GH)–inulin-like growth factor 1 (IGF-1) signaling to modulate the process of propagation of inflammaging. ↓, stimulation; ⊥, inhibition. Dotted lines of ↓ and ⊥ represent attenuation of stimulation and inhibition, respectively. Abbreviations in the figure: FoxA1 (Forkhead Box A1), Rb1 (RB Transcriptional Corepressor 1), IRF7 (interferon regulatory factor 7), IGF-IR (insulin-like growth factor 1 receptor), GHR (growth hormone receptor)

Retrotransposons of the long interspersed nuclear element 1 (Line-1 or L1) family generate genomic diversity with a copy-and-paste mechanism to propagate through RNA intermediates [70]. L1 retrotransposons generally encode a reverse transcriptase and endonuclease within the same open reading frame and are thought to be transcribed by RNA polymerase II [70]. They create new insertions and exert an enormous variety of structural and functional impacts on genes and genomes, which may cause diseases in mammals as well as genomic diversity. In normal cells, the transcriptional activity of L1 and its mRNA and cDNA are repressed by RB transcriptional corepressor 1 (Rb1) and three prime repair exonuclease 1 (Trex1). However, in senescent cells, the activities of Rb1 and Trex1 are decreased, whereas FoxA1 binds to L1’s 5’UTR and promotes its transcription, resulting in the accumulation of L1 elements [71]. These alterations induce type 1 interferon (IFN-I) response and then SASP in senescent cells (Figure 1). Although there is a lack of direct data from senescent cell models, the antiviral IFN-I response is reported to be negatively regulated by FoxO3 via targeting interferon regulatory factor (IRF)7 in macrophages [72,73] and in fibroblasts by acting as a gene repressor [74]. 

GH is not only secreted from anterior pituitary cells but is also expressed in various cells including immune cells [75]. Receptors for GH and IGF-1 are also expressed in macrophages, implying that GH and IGF-1 act on macrophages in a paracrine and/or autocrine fashion. Spadaro et al. [76] reported augmented sensitivity of macrophages derived from aged mice for activation of the NLRP3 inflammasome. The activation of inflammasome was inhibited in long-lived GH receptor (*Ghr*) gene-deleted mice [76], suggesting the involvement of GH–IGF-1 signaling in activation of the inflammasome. Because FoxOs are known to be inhibited by GH–IGF-1 signaling, we may surmise that FoxO3 inhibits the priming or activation of the inflammasome in macrophages, or both (Figure 1).

Based on the accumulated evidence described above, we hypothesize that CR exerts its salutary effects via attenuation of the activation of inflammasomes by FoxO-dependent mechanisms, particularly in a FoxO3-dependent manner. However, there is little evidence indicating the roles of FoxOs in activation of the inflammasome under CR conditions.

## 6. MicroRNA (miRNAs) in Inflammasomes and Aging

MicroRNAs (miRNAs) are noncoding RNAs (ncRNAs) of approximately 22 nucleotides that regulate target mRNAs at the post-transcriptional level by mediating their degradation and/or inhibiting their translation [77]. For mature miRNAs, one strand of the miRNA duplex is incorporated into the RNA-induced silencing complex (RISC), of which an Argonaute (Ago) protein is an essential component. The miRNA−RISC complex binds to the 3′-untranslated (UTR) region of a target mRNA, resulting in its degradation or repressed translation [78,79]. miRNAs are being intensively investigated as key regulators of target genes and as potential biomarkers in many areas within the fields of physiology and pathology [80,81,82,83]. 

Functional miRNAs are incorporated into Argonaute (Ago) family proteins (Ago1 to 4), which are ubiquitously expressed. Because Ago2 is the most highly expressed Ago protein in mice and has miRNA silencing activity [84], it is important to be able to isolate Ago2–miRNA complexes. Significantly improved detection sensitivity was shown for Ago-bound miRNAs compared with that of nonfunctional miRNAs that were not bound to an Ago protein [85]. Using this method with next-generation sequencing, we identified many Ago2-bound miRNAs expressed in murine skin wounds (miR-147, miR-129-3p, miR-139-5p, miR-21, and miR-340-5p) [78,86]. Subsequently, we selected Ago2-bound miRNAs for which there was definitive evidence of an association with inflammation (i.e., miR-139-5p, miR-223, miR-142-3p, and miR-142-5p) by comparing miRNA expression profiles of wild-type mice with those of *Spi1*-knockout mice displaying no inflammatory response because of a lack of immune cells [86]. It is speculated that there are a large number of miRNAs in a single cell, so Ago-bound miRNA requisition is important for the spatiotemporal functions of miRNAs associated with inflammasomes and age-related diseases. For example, middle-aged miR-223-deficient mice exhibited inflammatory lesions in the lung as a consequence of neutrophil hyperactivity [87]. Recently, Xu and colleagues also reported that miR-223 plays a protective role in neutrophilic asthmatic mice through the inhibition of NLRP3 inflammasome [88]. 

Many miRNAs have been identified to be involved in inflammaging, cellular senescence, and cancer, although this depends on the cell type [89]. To date, several miRNAs have been shown to be common to senescence and inflammasomes. For example, miR-9 regulates the expression of SIRT1, a longevity gene, which exhibits nicotinamide adenine dinucleotide (NAD)-dependent deacetylase activity, during murine embryonic stem cell differentiation and in adult murine tissues [90]. The activities of FoxOs are also modulated by deacetylation. miR-9 is reported to inhibit the transcription of NF-ĸB1 in human polymorphonuclear leukocytes and macrophages [91]. In addition, miR20a, which is downregulated during aging in humans [92], modulates the protein expression of apoptosis signal-regulating kinase (ASK) in lipopolysaccharide (LPS)-stimulated fibroblast-like synoviocytes derived from patients with rheumatoid arthritis [93]. Moreover, miR-21 acts to downregulate phosphatase and tensin homolog (PTEN), activate protein kinase B alpha (AKT), and increase NF-κB activation in LPS-stimulated macrophages. In those macrophages, miR-21 negatively regulates programmed cell death 4 (PDCD4), leading to the activation of NF-κB [94]. Circulating blood levels of miR-21 were found to be markedly increased in elderly people and patients with cardiovascular diseases, suggesting that miR-21 acts as a biomarker of inflammaging [95]. Furthermore, high levels of onco-miR-21 contribute to the senescence-induced growth arrest in normal human cells, whereas its knockdown increases the replicative lifespan [96]. Another study showed that miR-29 regulates B-Myb expression during cellular senescence in the HeLa cell line [97]. miR-21 and miR-29a were also found to exert their functions by binding as ligands to receptors of murine TLR7 and human TLR8 in immune cells [98]. miR-126 modulates inflammatory activities by downregulating the expression of IĸB-a, an important inhibitor of the NF-κB signaling pathway [99]. miR-126 was also reported to target TOM1 (target of Myb1), which has been shown to interact with the TLR2 and TLR4 signaling pathways, forming a complex to regulate the endosomal trafficking of ubiquitinated proteins [100]. miR-126 is also deregulated in several disorders characterized by endothelial cell activation in response to systemic inflammatory stimuli; such disorders include cardiovascular diseases, diabetes mellitus, and inflammatory diseases [99,101,102]. Upregulation of miR-146a, which binds to and downregulates IRAK1 and TRAF6, results in the reduction of NF-κB activity [103]. Olivieri and colleagues have reported that increasing miR-146a expression induced replicative senescence in human umbilical vein cells and in aortic and coronary endothelial cells, suggesting that miR-146a is a candidate marker of senescence-associated pro-inflammatory status in vascular remodeling [104]. miR-146a has also been identified as a mitochondrially associated miRNA that is involved in cellular senescence and inflammaging [105]. miR-155 is also known to be common to cellular senescence and inflammasomes [89,106]. Recently, Ekiz and colleagues reported that whole-body miR-142a-knockout mice exhibited a shortened lifespan. In addition, T cell-specific deletion of miR-155 in whole-body miR-146a-knockout mice suppressed the autoimmune phenotype and significantly extended their lifespan. Taking these findings together, many miRNAs are known to be involved in cellular senescence and inflammation; it should be noted that miR-146a and miR-155 exhibit critical roles in age-mediated immune dysfunction, and possibly influence lifespan [107]. 

As for the potential role of miRNAs in the regulation of FoxO3, miR223 was reported to inhibit the expression of FoxO3 in a viral infection model [72]. Another viral infection model also showed an inhibitory effect of miR-155 on the expression of FoxO3 [108]. miR223 and miR155 are upregulated by viral infection, leading to the suppression of FoxO3. Because FoxO3 inhibits interferon regulatory protein (IRF)-7, upregulation of those miRNAs consequently enhances type 1 interferon (INF-I) response (Figure 1). By contrast, in airway epithelial cell-specific *Foxo3* knockout mice, the IFN-I response to rhinovirus infection was shown to be reduced, suggesting the need for FoxO3 in the proper activation of innate immunity [109]. As described above, many miRNAs are involved in the regulation of inflammatory responses including inflammasome activation, but the regulatory circuits of the FoxO3–IFN-I response under conditions of CR remain to be investigated.

## 7. Development of an Assay and Mouse Models for Testing the Hypothesis

**Figure 2:**
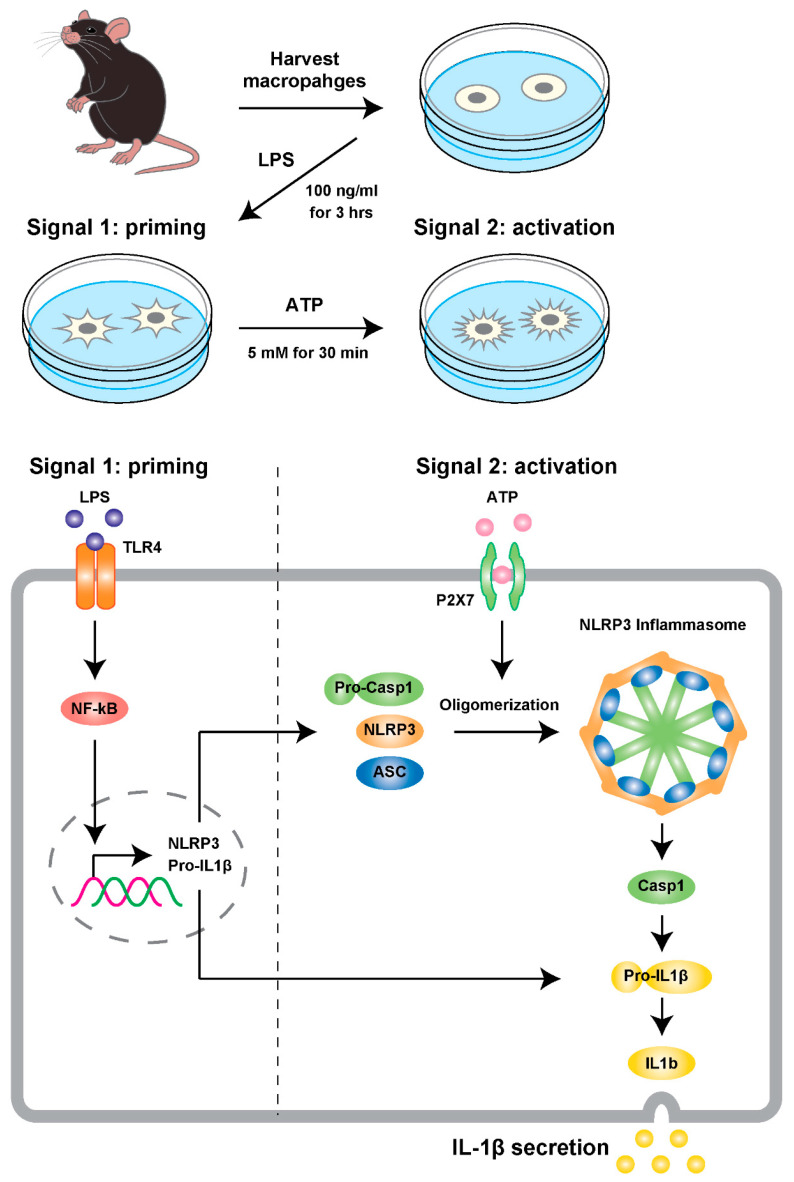
An experimental model of NLRP3 inflammasome activation using primary macrophages. A cell-based inflammasome activation model was briefly modified from a previously described approach [110]. In short, peritoneal exudate macrophages were harvested from the peritoneal cavity of mice 2 days after the intraperitoneal (i.p.) injection of 2 mL of 4% thioglycolate medium. The cells were cultured in RPMI 1640 medium containing 8% FBS, 100 units/mL penicillin, and 100 µg/mL streptomycin under a 5% CO_2_ atmosphere at 37 °C for 30 min. Prior to the assays, cells were washed twice with phosphate buffered saline (PBS) and further cultured for 3 h in Opti-MEM I Reduced-Serum Medium containing 100 ng/mL of lipopolysaccharides (LPS), which was the priming stimulus to induce pro-IL-1β transcription. Then, cells were treated with 5 mM ATP for 30 min to activate the inflammasome process, involving the secretion of IL-1β and the induction of inflammatory responses. Abbreviations in figure: TLR4 (toll-like receptor 4), P2X7 (Purinergic Receptor P2X, Ligand Gated Ion Channel, 7), ASC (Apoptosis-associated speck-like protein containing a CARD)

First, to determine whether CR alters the NLRP3 inflammasome activation in macrophages, we used a modified cell-based experimental model [110] (Figure 2). To assess the effects of CR, we measured the activation of caspase-1 and secretion of IL-1β in macrophages harvested from thioglycolate-stimulated peritoneal exudate cells (PEC macrophages; described in Appendix A). PEC macrophages were collected from seven- to eight-month-old male mice in the CR group or the AL group. CR, involving a 30% restriction of food intake, was initiated at 12 weeks of age. Collected PEC macrophages were primed with lipopolysaccharide (LPS) and subsequently stimulated by ATP for 30 min. ATP treatment activates caspase-1 by self-cleavage in PEC macrophages, and then the active form of caspase-1 converts pro-IL-1β to the active form IL-1β, which is released from PEC macrophages to the supernatant in culture.

**Figure 3:**
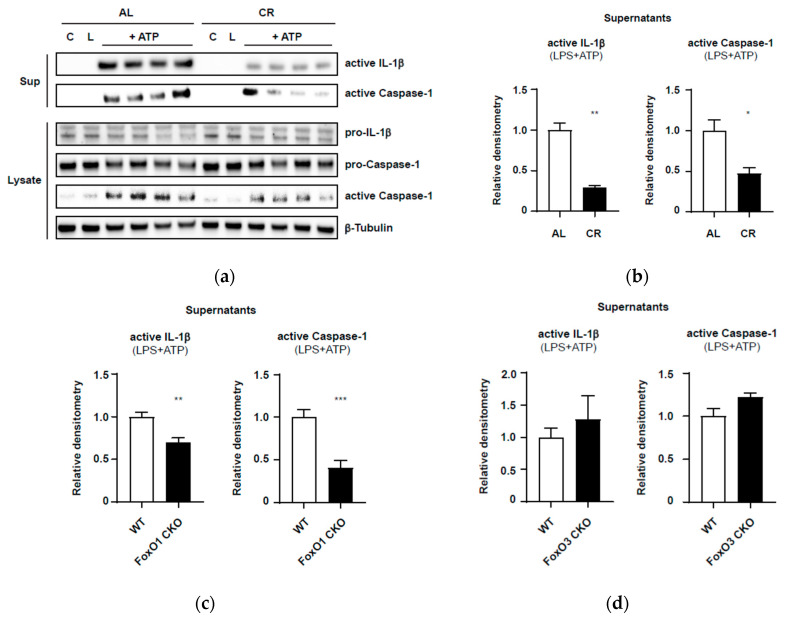
Activation of NLRP3 inflammasome in macrophages. (**a**) Representative immunoblots in peritoneal exudate (PEC) macrophages isolated from wild-type (WT) ad libitum (AL) and calorie restricted (CR) mice at 7–8 months of age. Cell lysates were used for immunoblotting of pro-IL-1β and pro-Caspase-1. Supernatants (Sup) were used for immunoblotting of active IL-1β and active Caspase-1. β-Tubulin was used as a loading control. (**b**) Densitometric analysis of immunoblots in the AL and CR groups of WT mice. The data represent the expression levels of active IL-1β (left panel) and active Caspase-1 (right panel) in supernatants in response to lipopolysaccharide (LPS) and ATP treatment. The densitometric values are normalized by β-tubulin expression. The results are presented as mean ± SEM (*n* = 4, * *p* < 0.05, ** *p* < 0.01, ratio to the AL group, Student’s t-test with Welch’s correction). (**c**) Densitometric analysis of immunoblots in macrophages isolated from the AL groups of WT and *Foxo1*-CKO (conditional knockout) mice. The results are presented as mean ± SEM (*n* = 7, ** *p* < 0.01, *** *p* < 0.001, ratio to WT group, Student’s t-test with Welch’s correction). (**d**) Densitometric analysis of immunoblots in macrophages isolated from the AL groups of WT and *Foxo3*-CKO mice. The results are presented as mean ± SEM (*n* = 4, ratio to WT group, Student’s t-test with Welch’s correction).

As shown in Figure 3a,b, PEC macrophages from the CR mice showed reduced expression of the active forms of caspase-1 and IL-1β in the supernatant, compared with PEC macrophages from AL mice. Moreover, CR significantly suppressed the cleavage of pro-caspase-1 in the cell lysates, compared with AL. These results indicate that CR attenuates activation of the NLRP3 inflammasome in response to DAMPs even in relatively young mice (7–8 months old).

This preliminary finding is in accordance with a previous in vivo study that reported that CR reduced inflammation and enhanced insulin sensitivity with the suppression of IL-1β and NLRP3 expression in a type 2 diabetes mouse model [57]. Because PEC macrophages harvested from CR mice retained the expected CR phenotypes in vivo, our modified assay for activation of the NLRP3 inflammasome in the primary culture may be used to evaluate the inflammasome activation in PEC macrophages harvested from aging mice or from mice with conditional knockout of the *Foxo1* or *Foxo3* gene.

Next, to identify possible roles of FoxO1 and FoxO3 in activation of the inflammasome under CR conditions, we established mice with conditional knockout of the *Foxo1* (*Foxo1*-CKO) or *Foxo3* gene (*Foxo3*-CKO) specifically in the myeloid cell lineage (including monocytes, macrophages, and granulocytes) using the Cre-LoxP system (Appendix A.1 and Supplemental Figure S3). As the first step, we confirmed the activation of the NLRP3 inflammasome in the presence or absence of FoxOs in PEC macrophages in AL mice. As shown in Figure 3c and Supplemental Figure S2a, compared with those in the WT group, PEC macrophages from *Foxo1*-CKO mice showed significant reductions in ATP-induced caspase-1 activation and active IL-1β secretion in supernatants. Meanwhile, PEC macrophages isolated from *Foxo3*-CKO mice demonstrated no difference in caspase-1 activation or active IL-1β release in response to ATP compared with that in WT mice (Figure 3d; Supplemental Figure S2b). These preliminary experiments suggested differential roles for FoxO1 and FoxO3 in inflammasome activation.

It should be noted that these preliminary results in PEC macrophages were obtained from mice fed AL at a young age. As reported in our lifespan studies, haploinsufficiency of the *Foxo1* or *Foxo3* gene did not affect the lifespans and incidences of neoplasms at death in the AL groups [51,68]. This means that only a 50% reduction of FoxO1 or FoxO3 expression does not affect development, growth, and aging in mice under standard husbandry conditions, namely, specific-pathogen-free and AL feeding conditions. In our murine skin wound model, *Foxo1*^+/−^ AL mice showed more improved skin wound healing and scarring than WT-AL mice [111]. In *Foxo1*^+/−^ AL mice, both dermal inflammation and granulation tissue formation were shown to be attenuated. However, AL-fed mice with homozygotic deletion of FoxO3 (*Foxo3*^−/−^) did not show any difference in the wound healing process compared with AL-fed WT mice [111]. The attenuation of activation of the NLRP3 inflammasome in *Foxo1*-CKO macrophages may be in line with the findings of our skin wound study in *Foxo1*^+/−^ AL mice. However, in the AL feeding conditions, the attenuation of inflammasome activation in macrophages may not be effective enough to inhibit tumors and prolong lifespan in mice. The differential roles of FoxO1 and FoxO3 emerged under CR conditions [51,68]. Potential roles for FoxOs could be context-dependent; in particular, interactions between nutritional conditions and FoxO isoforms have been identified. 

## 8. Perspective

Inflammaging or senoinflammation, which could be elicited by activation of the NLRP3 inflammasome, is a compelling hypothesis in aging research. Processes of inflammasome activation are targets of therapies for aging-related diseases. With regard to the effects of CR, it remains to be elucidated whether CR attenuates processes of inflammasome activation in myeloid cells and diminishes insults surrounding parenchymal cells, or whether CR strengthens protective mechanisms against persistent inflammation in aging tissues, or both. The presence of the isoform-specific contributions of FoxO1 and FoxO3 to the tumor-inhibiting and life-extending effects of CR in mice warrants future studies using mice with myeloid-cell-lineage-specific deletion of the *Foxo1* or *Foxo3* gene under CR conditions. These mouse models could elucidate molecular mechanisms by which FoxO1 and FoxO3 differentially regulate neoplastic and aging processes under CR conditions from the perspective of inflammaging.

## Supplementary Materials

The following are available online at https://www.mdpi.com/2072-6643/12/7/1959/s1, Figure S1: Brain weights in male mice in middle age (9–12 months of age); Figure S2: Immunoblots of active IL-1β and active caspase-1 in supernatants and cell lysates of macrophages; Figure S3: Immunoblots of FoxO1 and FoxO3 in macrophages from *Foxo*1-CKO and *Foxo3*-CKO mice.

## Appendix A

### Appendix A.1. Calorie-Restricted Mouse Model and Generation of Myeloid-Lineage-Specific Foxo1 and Foxo3a Conditional Knockout Mice

All experiments were conducted in accordance with the provisions of the Ethics Review Committee for Animal Experimentation at Nagasaki University. Mice were maintained in a barrier facility (temperature; 22–25 °C, 12-h light/dark cycle) under specific-pathogen-free conditions. Mice were fed ad libitum with Charles River-LPF diet (360 kcal/100 g: 13% fat calories, 26% protein calories, and 61% carbohydrate calories (Oriental Yeast, Tokyo, Japan)). AL-fed and CR-fed C57/BL6/J mice were raised as follows, as also described previously [51,59]. Briefly, the mice were divided into the AL and CR groups at 12 weeks of age. The CR group was provided with a food allotment consisting of 70% of the mean daily food intake of the AL group. The food allotments for the CR groups were adjusted depending on the amount of food consumed by the AL groups every 2 weeks, until 30 weeks of age. Body weight was monitored every 2 weeks between 8 and 30 weeks of age.

LysM-Cre (RBRC02302, RIKEN) [112], *Foxo1^fl^*^/*fl*^, and *Foxo3^fl^*^/*fl*^ mice were purchased from Jackson Laboratories and generated as described previously. To generate male myeloid-lineage-specific *Foxo1* and *Foxo3* conditional knockout mice, male *LysM*-*Cre* mice were crossed with female *Foxo1^fl^*^/*fl*^ and *Foxo3^fl^*^/*fl*^ mice to produce *LysM*-*Cre* heterozygous-expressing *Foxo1^fl^*^/+^ mice and *Foxo3^fl^*^/+^ mice. A second cross-generated male *LysM*-*Cre* heterozygous-expressing *Foxo1^fl^*^/*fl*^ mice and *Foxo3^fl^*^/*fl*^ mice. Male *LysM*-*Cre*::*Foxo1^fl^*^/*fl*^ and male *LysM*-*Cre*::*Foxo3^fl^*^/*fl*^ mice were crossed with female *Foxo1^fl^*^/*fl*^ and *Foxo3^fl^*^/*fl*^ mice to produce control mice (*LysM*^+/+^::*Foxo1^fl^*^/*fl*^ mice, *LysM*^+/+^::*Foxo3^fl^*^/*fl*^ mice) and conditional knockout mice (*LysM*-*Cre*::*Foxo1^fl^*^/*fl*^ mice, *LysM*-*Cre;Foxo3^fl^*^/*fl*^ mice) as litter. We used littermates in all of the experiments. Mouse genotypes were defined by PCR, as previously described [53].

### Appendix A.2. Cell-Based NLRP3 Inflammasome Model

#### Appendix A.2.1. PEC Macrophage Isolation and NLRP3 Inflammasome Activation Model

Peritoneal exudate cell (PEC) macrophages were collected from 30-week-old AL and CR mice, 2 days after 4% thioglycolate (BD Diagnostic Systems, Heidelberg, Germany) was given by i.p. injection. Collected PECs were strained with a 40 μm mesh strainer, after which the cells were counted with Countess (Thermo Fisher Scientific, Waltham, MA, USA). Isolated PECs were seeded into 12-well plates at 3 to 5 × 10^6^ cells/well and then incubated with RPMI 1640 medium with 8% FBS and 1% penicillin/streptomycin (Thermo Fisher Scientific) for 30 min. Attached cells were washed twice with culture medium and PEC macrophages were primed with 100 ng/mL LPS (Sigma Aldrich, Germany) for 3 h. After LPS priming, cells were washed three times with Opti-MEM I Reduced-Serum Medium (Thermo Fisher Scientific), after which the primed cells were exposed to 5 mM ATP (Wako, Tokyo, Japan) for 30 min in Opti-MEM medium. After 30 min, supernatants were taken for the measurement of active IL-1β and caspase-1. In addition, cell lysates were collected for determining the premature forms of IL-1β and caspase-1. β-Tubulin was used as a loading control.

#### Appendix A.2.2. Bone Marrow-Derived Macrophage Isolation

Bone marrow (BM)-derived macrophages were isolated by MACS cell separation technology with Microbeads kit (Miltenyi Biotec Inc., Bergisch Gladbach, Germany) as following manufacturer’s instructions. Cells were reacted with anti-F4/80 Ab to isolate BM-derived macrophages [86].

#### Appendix A.2.3. Immunoblot Analysis

For the immunoblot analysis of culture supernatant, culture medium was taken and centrifuged at 400× *g* for 2 min. The supernatant was transferred to a new microtube to which the same volume of methanol was added, followed by mixing robustly. In addition, one-quarter volume of chloroform was added and mixed robustly, followed by incubation on ice for 15 min. After incubation, samples were centrifuged at 20,000× *g* for 10 min at 4 °C, after which the upper phase of the sample was removed. A total of 500 μL of methanol was added to the remaining sample and mixed robustly, followed by incubation on ice for 15 min. After incubation, samples were centrifuged at 20,000× *g* for 10 min at 4 °C and the supernatant was then removed. Subsequently, 500 μL of fresh methanol was added alone, without mixing, after which the sample was centrifuged again the same as mentioned above. After the supernatant had been clearly removed, the remaining pellet was briefly air-dried. The pellets from the supernatant and harvested cells were lysed with Tissue Protein Extraction Reagent (T-PER, Thermo Fisher Scientific) containing protease and phosphatase inhibitor cocktail (Thermo Fisher Scientific). Supernatant and cell lysate samples were separated on 4–12% NuPAGE Novex Bis-Tris gels (Invitrogen) and transferred to a PVDF membrane using an iBlot gel transfer device (Invitrogen). Protein bands were visualized using chemiluminescence detection reagent, Immunostar LD (Wako). Images of immunoblotting were taken by Fusion Solo S (Vilber, Marne-la-Vallee, France). The data were analyzed using Multi Gauge software (Fujifilm, Tokyo, Japan). The following monoclonal or polyclonal antibodies (mAbs or pAbs) were used for immunoblotting: rabbit anti-cleaved IL-1β pAb (MBL, Nagoya, Japan), mouse anti-caspase-1 (p20) mAb (Adipogen, San Diego, CA, USA), rabbit anti-FoxO1 mAb (Abcam, Cambridge, UK), rabbit anti-FoxO3 mAb (Cell Signaling Technology, Danvers, MA) and rabbit anti-Gapdh pAb (Abcam), rabbit anti-β-tubulin pAb (Abcam, Cambridge, UK). Horseradish peroxidase (HRP)-conjugated anti-rabbit or anti-mouse IgG mAbs (GE Healthcare Life Sciences, NJ, USA) were used as secondary antibodies.
