# Supplementary material for: Does Calorie Restriction Modulate Inflammaging via FoxO Transcription Factors?
Source: Nutrients. 2020 Jun 30;12(7):1959. doi: 10.3390/nu12071959 (PMC7399912; doi:10.3390/nu12071959)
Supplement: Supplementary file 1 [file nutrients-12-01959-s001.zip › nutrients-841364-supplementary/Supplemental data v2.docx]

**Supplemental Information**

**Figure S1**. Brain weights in male mice in middle age (9–12 months of age). The bars and lines represent the means and standard deviations (SD: n = 7 to 11 for each group). ** p < 0.001 vs. WT-AL, Foxo3^+/−^ AL, Foxo3^+/-^ CR, and Foxo3_1^+/−^ CR by Tukey’s honestly significant difference (HSD) test.

**
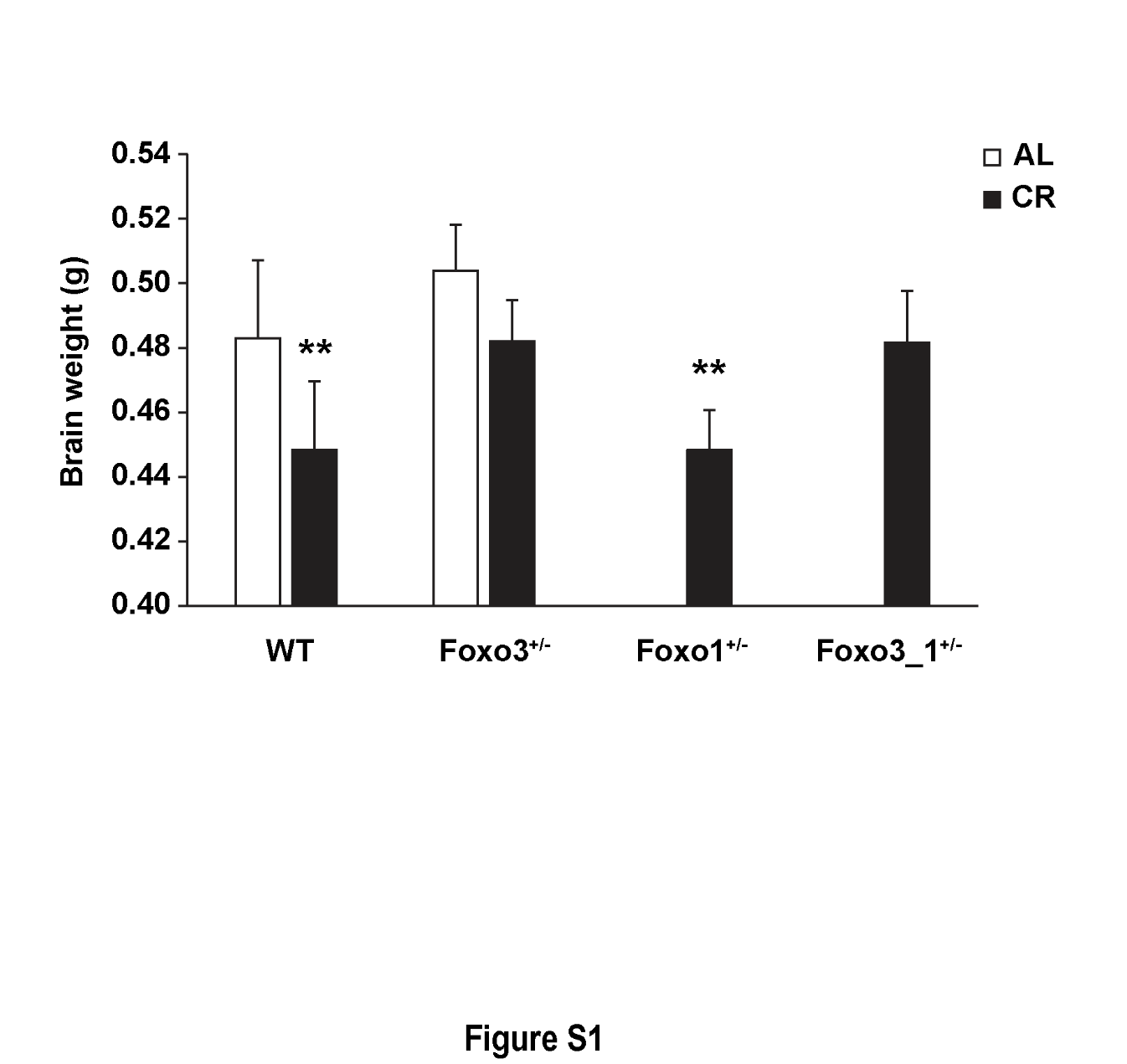
**

**
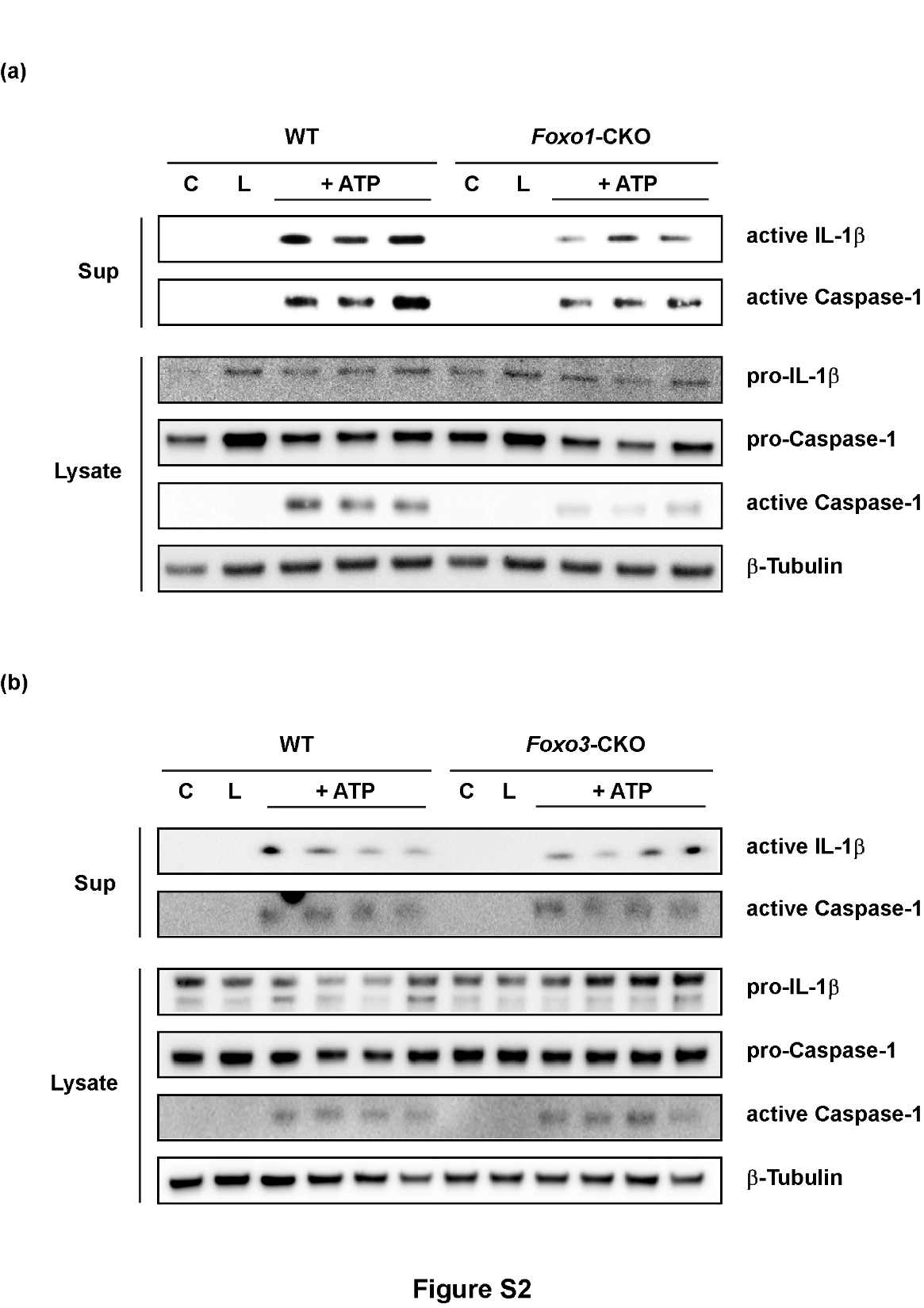
Figure S2**. FoxO1, but not FoxO3, attenuates activation of NLRP3 inflammasome in macrophages. Conditional Foxo1 or Foxo3 gene knockout (*Foxo1*-CKO or *Foxo3*-CKO) regulates NLRP3 inflammasome activation in peritoneal macrophages. (a) Immunoblot analysis of active IL-1β and active Caspase-1 in PEC macrophages were isolated from WT and *Foxo1*-CKO mice. (b) Immunoblot analysis of PEC macrophages were isolated from WT and *Foxo3*-CKO mice. The expression of β-Tubulin was used as a loading control.

**
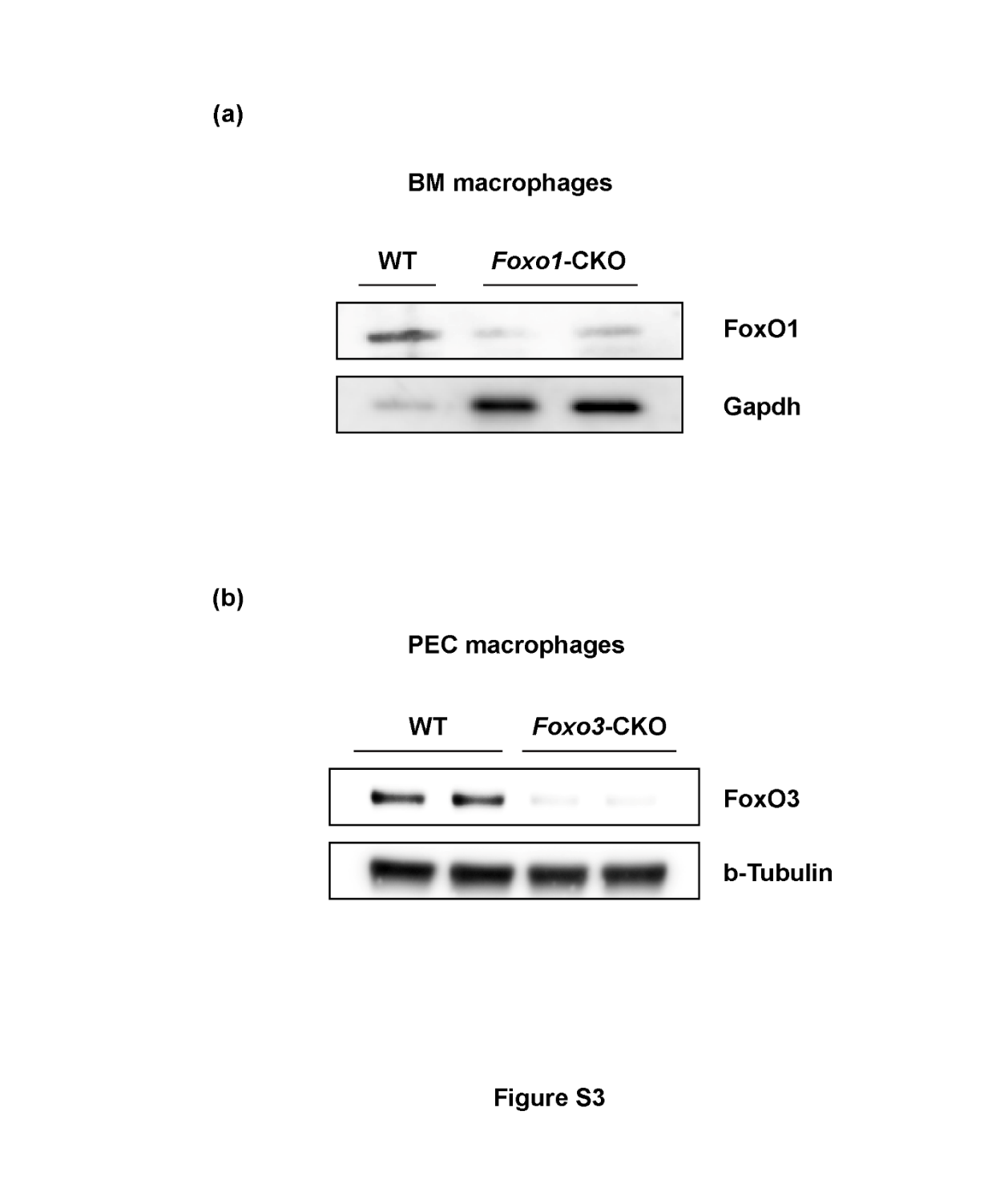
Figure S3.** Immunoblotting for determining expression level of FoxO1 and FoxO3 in primary macrophages derived from *Foxo1*-CKO and *Foxo3*-CKO mice, respectively. (a) The expression level of FoxO1 in BM macrophages from WT and *Foxo1*-CKO mice. The expression of Gapdh was used as a loading control. (b) The expression level of FoxO3 in PEC macrophages from WT and *Foxo3*-CKO mice. The expression of β-Tubulin was used as a loading control.
