# Supplementary figures and images for: Does Calorie Restriction Modulate Inflammaging via FoxO Transcription Factors?
Source: Nutrients. 2020 Jun 30;12(7):1959. doi: 10.3390/nu12071959 (PMC7399912; doi:10.3390/nu12071959)

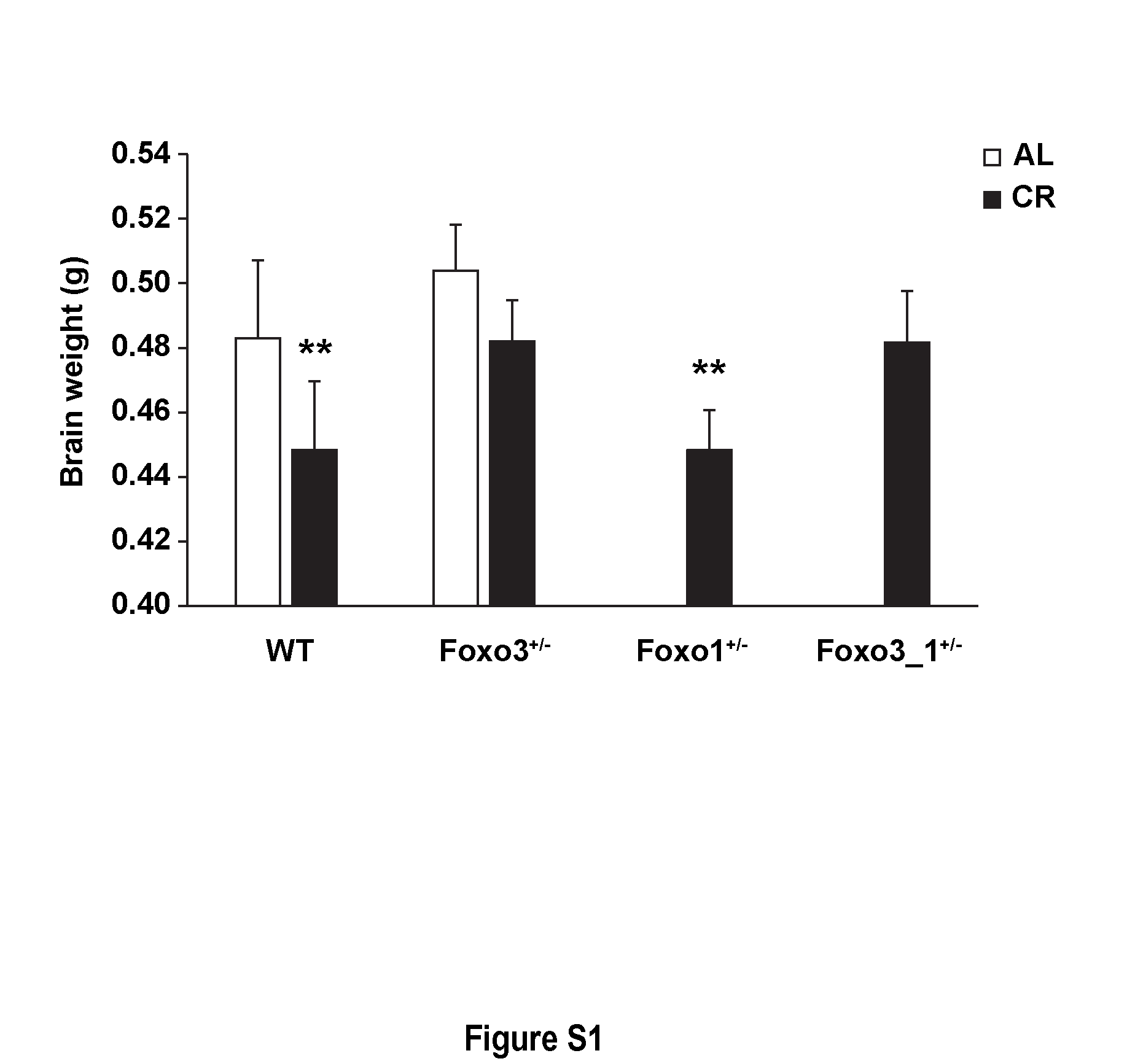

Supplement: Supplementary file 1 [file nutrients-12-01959-s001.zip › nutrients-841364-supplementary/Supplemental figure S1.tif]

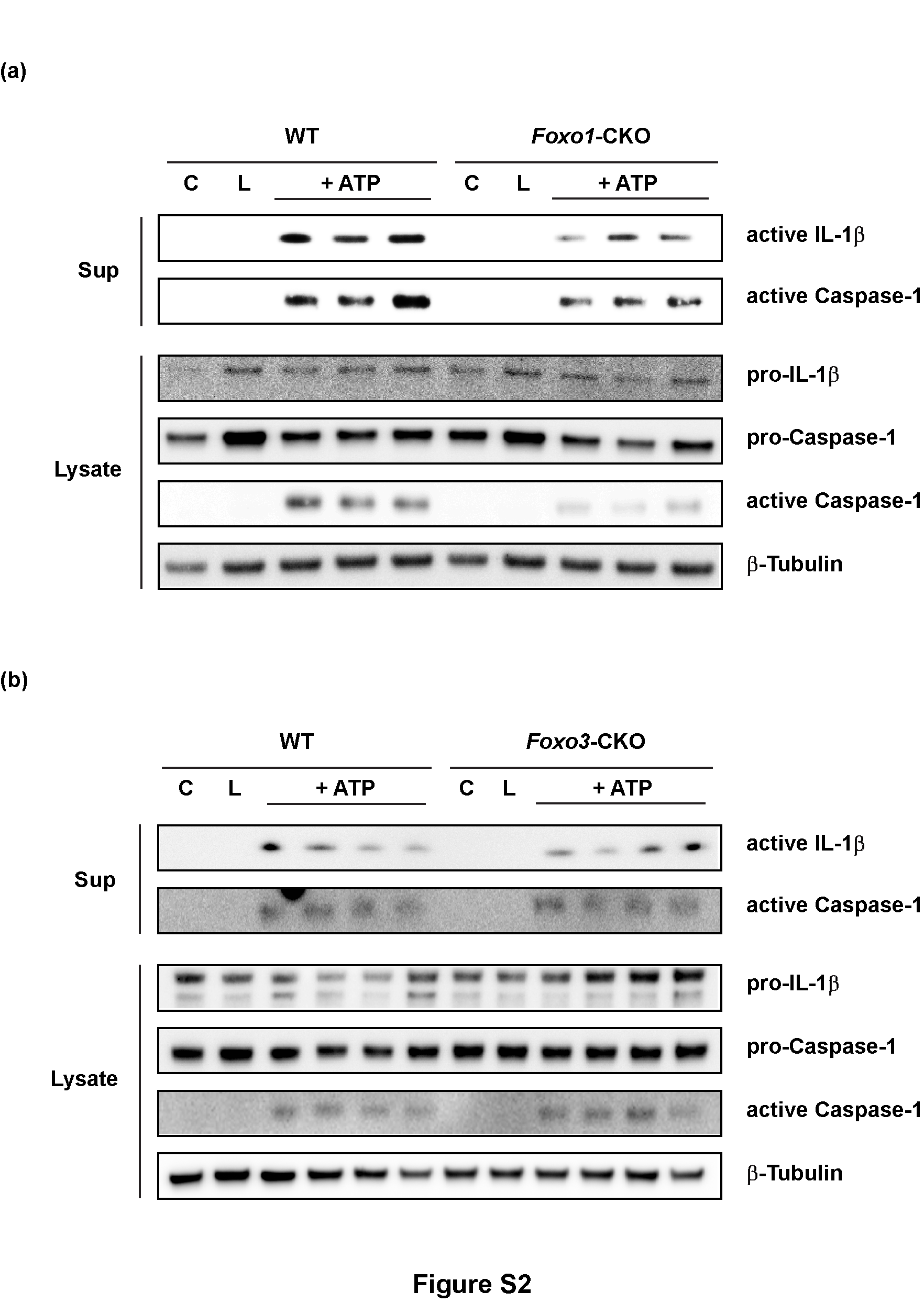

Supplement: Supplementary file 1 [file nutrients-12-01959-s001.zip › nutrients-841364-supplementary/Supplemental figure S2.tif]

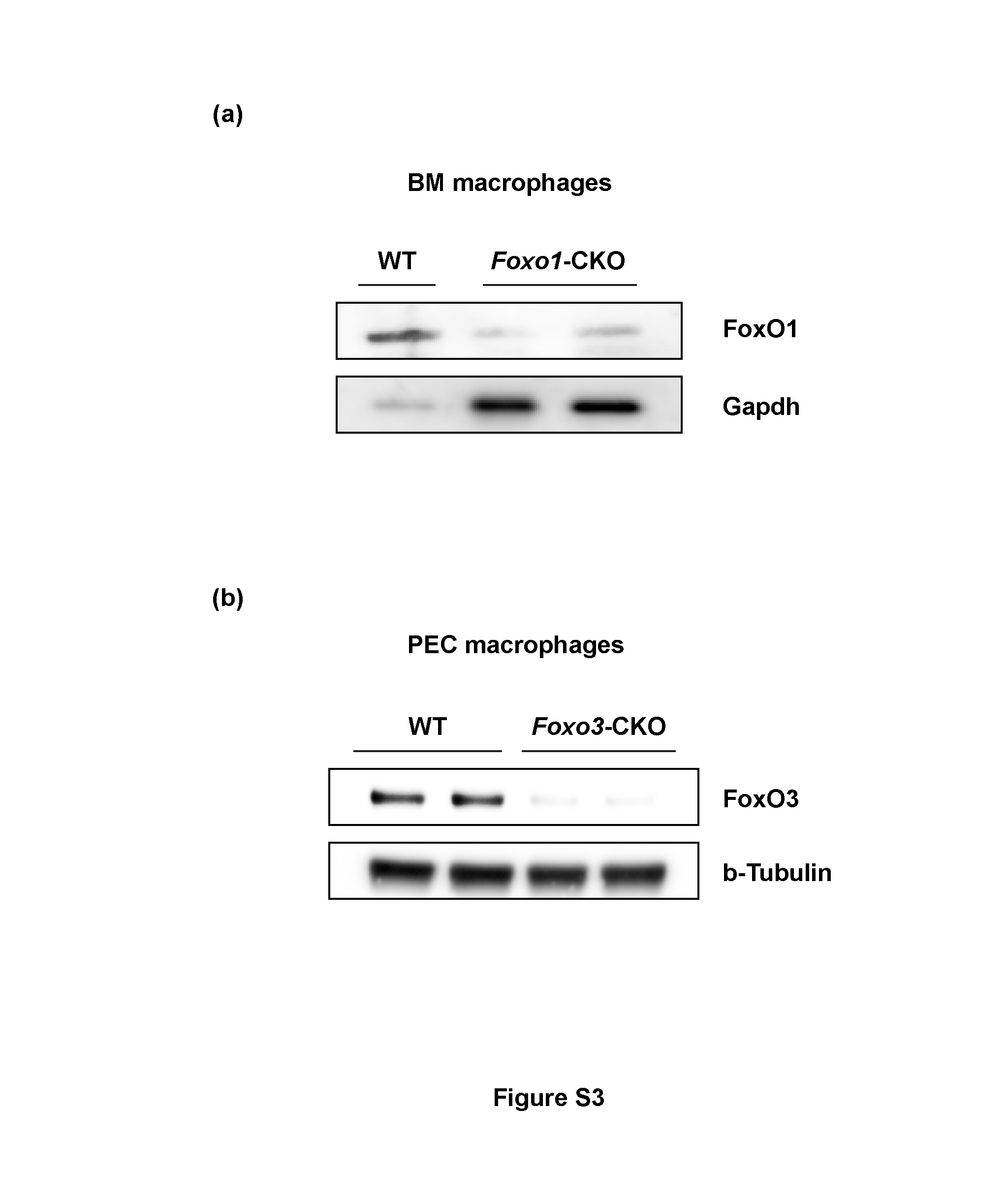

Supplement: Supplementary file 1 [file nutrients-12-01959-s001.zip › nutrients-841364-supplementary/Supplemental figure S3.tif]
